# Digital Addiction Scale for Children (DASC): Age, Gender, Sleep and Emotional Correlates

**DOI:** 10.5964/ejop.17937

**Published:** 2026-02-27

**Authors:** Elena García-Morales, Cristina Cuesta-Zamora, Fernán Arana, Natalia Olmeda, Alberto Tapia-Bernal, Jorge Javier Ricarte

**Affiliations:** 1 Department of Psychology, University of Castilla-La Mancha, Albacete, Spain; 2 Department of Counseling and Psychological Services, University of Buenos Aires, Buenos Aires, Argentina; Birmingham City University, Birmingham, United Kingdom

**Keywords:** digital addiction, sleep, anxiety, depression, children

## Abstract

Nowadays, digital devices (DD) overuse is an increasing risk factor to develop anxiety, depression and sleep disturbances in young population. **Objectives:** This study aims to validate the Digital Addiction Scale for Children (DASC) in Spanish, the second most spoken language globally, and to examine its relationship with anxiety, depression, and sleep patterns in children and adolescents. **Methods:** A sample of 977 children aged 9 to 14 completed self-reported measures of sleep duration, the Patient-Reported Outcomes Measurement Information System (PROMIS) and the DASC. The statistical analyses, including confirmatory factor analysis, Cronbach’s alpha, McDonald’s omega, and Pearson correlations, revealed that the DASC has a bifactorial structure and good reliability. **Results:** Higher problematic DD levels were associated with increased anxiety and depression symptoms and reduced sleep hours. Although the DASC-Spanish version shows only partial gender and age invariance, it demonstrates strong psychometric properties, supporting its use in psychological practice and research. **Conclusions:** The observed correlates of the Spanish-DASC with anxiety, depression, and sleep; emphasize its relevance in the presence of negative emotional symptoms and for the promotion of psychological well-being and health in late childhood and adolescence.

## Digital Addiction Scale for Children (DASC): Age, Gender, Sleep and Emotional Correlates

The use of digital devices (DD), such as smartphones and computers, has markedly increased world-wide in the last decades (Ferrara et al., 2017). So much so that DD have been considered integral elements in children and adolescents’ everyday life (Christakis, 2019; Ferrara et al., 2017; Kaess et al., 2014). DD allow individuals to quickly find information, communicate and connect with others (Kaess et al., 2014). Nevertheless, in some cases, the use of DD and related activities like social media apps, watching videos or playing videogames, may predispose towards digital *addiction* (Christakis, 2019; Meng et al., 2022). Although digital addiction has not yet been included in the eleventh revision of the International Statistical Classification of Diseases (World Health Organization, 2017) and the fifth edition of the Diagnostic and statistical manual of mental disorders (DSM-5; American Psychiatric Association, 2013), evidence underscores that digital addiction is an emerging prevalent mental health condition (Christakis, 2019). In this line, the prevalence of smartphone addiction ranges between 15.19% to 19.61% in primary and secondary school students (Meng et al., 2022).

Several definitions of problematic DD use have emerged in the literature. An early conceptualization of problematic DD was the exceedance of the time recommended by experts, 2 hours (Tremblay et al., 2011). However, it is recognized that operationalizing problematic DD as exceedance of time alone is not enough to consider the experience as harmful (Christakis & Hale, 2025). Indeed, some evidence did not find significant associations between DD frequency and internet addiction (e.g., Stanković et al., 2021). The lack of associations between DD frequency and digital addiction may be explained by the fact that DD exposure is mandatory for school tasks or receiving social support (Christakis, 2019). Therefore, as highlighted in several models of digital addiction, it is important to consider not only the time spent using a DD, but also the biopsychosocial processes related to problematic DD use, such as symptoms of withdrawal or the DD interference in individuals’ lives (e.g., Almourad et al., 2020).

Given that the need to measure not only screen time, but also biopsychosocial processes related to problematic DD, along with the lack of measures to assess problematic DD use in children under 12 years old, Hawi et al. (2019) developed the 25-item *Digital Addiction Scale for Children* (DASC). The DASC was designed according to the DSM-5 Internet Game Addiction criteria and Griffiths’ addiction criteria. The DASC consists of two factors: interpersonal and intrapersonal (Bağatarhan & Siyez, 2023; Hawi et al., 2019). The 13 item DASC- Interpersonal factor, which involves whether DD may cause interferences in the child’s social dimension, measures four key elements of problematic DD:

1. *Deception* (referred to the lies related to DD use).
2. *Conflicts* (characterized by family arguments focused on limiting the DD use).
3. *Displacement* (which involves the loss of interest in hobbies and family activities due to the DD use).
4. *Problems* (focused on the uncontrollability of DD despite its interferences at school, sleep, family environment and money management).

The 12-item DASC-Intrapersonal factor involves five internal and individual processes of problematic DD:

1. *Preoccupation* (referred to concerns when unable to use the DD).
2. *Mood modification* (that assesses the use of DD as avoidance behavior to cope with displeasing emotions and experiences).
3. *Withdrawal* (that consists of uncomfortable emotions, such as guilt, frustration or anxiety, when unable to use the DD).
4. *Tolerance* (characterized by the need to increase the DD amount of time).
5. *Relapse* (refers to unsuccessful attempts to reduce and control the DD use).

DD are frequently used by children aged from 9–11 years old. Regarding age differences in relation to digital addiction, Lozano-Blasco et al. (2022) reported an inverse association between age and internet addiction in adolescents, suggesting that early adolescents may be a greater risk of internet addiction compared to late adolescents. It is important to mention digital addiction is a broader construct that includes behaviours beyond internet use, such as addiction to videogames, social networks and mobile phone. Despite the variety of DD, all digital addiction subtypes share similar brain responses and are associated with similar interpersonal impairments (Yang et al., 2024).

Sleep is a main indicator of well-being in children (Matricciani et al., 2019). Thus, it is relevant to know how DD use can affect sleep quality. Therefore, LeBlanc et al. (2015) found that most of 54.2% of 9–11-year-old children from nine countries use DD more than two hours per day. However, since exceedance of screen time alone is no longer considered a useful element to discriminate between problematic and not problematic DD use (e.g., Huang et al., 2023), further research is needed to determine whether children from 9–11 years old are at high risk for problematic DD. Furthermore, it is essential to develop validated tools to detect children that may be at risk for problematic DD use (Hawi et al., 2019). The DASC has been considered an instrument for screening problematic DD use in children and early adolescents in different languages, such as French (Hawi et al., 2019) or Turkish (Bağatarhan & Siyez, 2023; Çimke et al., 2023). However, this instrument has not been validated in Spanish, the second world-wide mother tongue, the second language with the most speakers and the third most translated language (Instituto Cervantes, 2024).

### Problematic DD, Anxiety, Depression and Sleep Duration

In late childhood and emerging adolescence, evidence suggests an increasing vulnerability to mental health disorders (Anderson et al., 2024) including anxiety, depression and sleep disturbances. DD have been considered a *powerful* technological tool that may affect individuals’ emotional sphere (Stanković et al., 2021). Accordingly, young individuals may use DD as a distraction strategy to avoid unpleasant emotions, such us symptoms of anxiety or depression (Hoge et al., 2017). Among children and adolescents, higher symptoms of anxiety and depression were linked to higher screen time (Maras et al., 2015) and problematic internet use (Lozano-Blasco & Cortés-Pascual, 2020; Restrepo et al., 2020). Nevertheless, evidence suggest that not all cases of DD use would be linked to worse symptoms of anxiety and/or depression (Hoge et al., 2017). For example, Restrepo et al. (2020) did not find significant differences on symptoms of anxiety between those with problematic internet use and those who did not manifest it. Similarly, Liu and colleagues (2016) found in a meta-analysis that those preadolescents that used DD less than two hours per day scored significantly lower in depression compared to those preadolescents who reported not using DD (Liu et al., 2016), suggesting that using DD less than 2 h/day may reduce the risk of depression. A plausible reason for the discrepancies between studies may be due to the content of the instruments used, which do not always cover the full heterogeneity of the behaviours implied in digital addiction. Most of the instruments employed in previous research focus solely on specific types of digital addiction, such as gaming or Internet use (Ding & Li, 2023). Besides, a considerable amount of previous research has focused on problematic Internet use (e.g., Lozano-Blasco & Cortés-Pascual, 2020; Restrepo et al., 2020), however, some DD can be used without internet access. On the other hand, some guidelines for children and adolescents suggest that less than 2 hours per day can have positive effects (e.g., LeBlanc et al., 2015). Therefore, it is possible to suggest that interpersonal and intrapersonal dimensions of problematic DD use, rather than DD frequency, may be more related to symptoms of anxiety and depression.

In addition, although the relationship between mood symptoms and problematic DD use has been deeply investigated in adolescents, research focused on preadolescent population is still in its infancy. To our knowledge, in children, only Hawi et al. (2019) has examined the links between problematic DD use (measured with the DASC) and symptoms of anxiety and depression. Hawi et al. (2019) found positive and significant correlations between problematic DD, and symptoms of anxiety and depression. However, the associations between both DASC dimensions and separate symptoms of anxiety and depression have yet to be explored in children.

Sleep pattern disturbances have demonstrated to be key underlying processes in the relationship between digital addiction and symptoms of depression (Karakose et al., 2023; Yuan et al., 2023). Sleep disturbances were significantly higher in children and adolescents with problematic internet use compared to those without problematic internet use (Restrepo et al., 2020). However, the links between sleep duration and problematic screen use have yet to be explored.

### Gender and Age Differences

Longitudinal research suggests significant differences in age and gender patterns of symptoms of depression, anxiety and problematic DD use from childhood to adolescence, highlighting the importance of considering age and gender differences in the studies. Regarding mood symptoms, Lewis et al. (2020) in a 10-year longitudinal study found that in the group that showed an increase in depressive symptoms between 4 and 14 years of age, females were at 80% greater risk of belonging to this group compared to males. Regarding problematic DD, digital addiction negatively correlates with age during adolescence (Lozano-Blasco et al., 2022). There is a significant link between screen time and depression in adolescents aged 14 years, although this link was not found in adolescents over 14 years old. Those results suggest that younger adolescents may be more vulnerable to DD use (Liu et al., 2016). Among children, Hawi et al. (2019) found gender differences in DD use. Males showed significantly higher symptoms of digital addiction than females. However, evidence is mixed. For example, Restrepo et al. (2020) did not find significant differences between males and females on problematic internet use.

While the consequences of problematic DD use (depression, anxiety and sleep disturbances) have been more extensively studied in adolescents (e.g., Mihara et al., 2016) than in children, recent research highlights that problematic DD use may be a hallmark in younger ages (Liu et al., 2016). Moreover, evidence suggests that most adult anxiety and depression disorders may have their roots in adolescence or even childhood (Jones, 2013), therefore, further research exploring the links between anxiety, depression and problematic DD use is needed in preadolescents. Based on all these arguments, this study explored whether the DASC two-factor structure (interpersonal and intrapersonal dimensions) found in the original instrument could be replicated in Spanish children. Simultaneously, this study examined the invariance across gender and age, the convergent validity with anxiety and depression and the association with hours of sleep.

Based on these objectives and the findings of previous research, five hypotheses were formulated:

- The original DASC two-factor structure will be confirmed in Spanish sample.
- The Spanish version of the DASC would exhibit accurate internal consistency.
- The Spanish version of the DASC would show no gender and age variances.
- Regarding the convergent validity, the Spanish version of the DASC would be significant and positively associated with anxiety and depression symptoms.
- Higher scores in the DASC would be associated with less amount of sleep hours.

## Method

### Participants

A total of 977 children (505 boys and 472 girls) with a mean age of 10.65 years (*SD*: 1.42; range: 9 to 14 years-old) participated in this study. Participants were recruited from 16 elementary schools in Castilla-La Mancha and Murcia (Spain). Participants ranged from the fourth grade of Primary Education to the third grade of Secondary Education. The majority, 84.5%, were children aged 9 to 11, while only 15.5% (*n* = 151) were preadolescents aged 12 to 14.

### Instruments

The *Patient Reported Outcomes Measurement Information System* (PROMIS, Cella et al., 2010) was employed to collect data on symptoms of depression and anxiety. Specifically, the PROMIS Anxiety Short Form and the PROMIS Emotional Distress — Depression Short Form were used, each consisting of eight items designed to assess participants’ cognitive and affective experiences of these symptoms. Responses are given on a Likert scale ranging from 1 (never) to 5 (always), with higher scores indicating a greater presence of anxiety and depression over the previous 7 days (Cella et al., 2010). Forrest et al. (2012) have validated the suitability of these measures for use with children and adolescents.

The *Digital Addiction Scale for Children* (DASC, Hawi et al., 2019) is a self-report measure created to assess level of addiction of digital devices in children. Participants have to rate the 25 items that composed the DASC on a Likert Scale of 5 points of frequency, being 1 “Never” and 5 “Always”.

A combined score is calculated by adding up all 25 items, resulting in a total between 25 and 125. Higher scores represent greater levels. The original DASC version reveals a robust two-factor structure, comprising interpersonal factors (Items 4, 16, 9, 22, 6, 18, 17, 20, 10, 13, 23, 25, 14) and intrapersonal factors (Items 1, 11, 19, 5, 15, 24, 3, 8, 12, 21, 2, 7). Additionally, it demonstrated excellent internal consistency with an alpha of 0.94 in children aged from 9 to 12 (Hawi et al., 2019). The translation process involved three researchers proficient in English from this study and an English native speaker. Firstly, a researcher translated the English-DASC version into Spanish. After that, two other researchers independently reviewed the translated Spanish-DASC version. Any questions were clarified in a meeting. The final Spanish version was back translated into English by a native English translator.

### Procedure

This study was approved by the Clinical Research Ethics Committee and strictly followed the latest version of the Declaration of Helsinki (World Medical Association, 2013), outlining ethical principles for research involving human subjects. A written document explaining the objectives, the anonymous nature of this research and the possibility to abandon the study at any time without explanations was given to the parents. Parents were provided with an email contact to ask for more information or clarifications. Parents and participants who agreed to participate in the study provided their written consent.

The questionnaires were administered collectively during school sessions and required approximately 25 minutes to complete. To begin with, children provided sociodemographic details (grade level in Primary or Secondary education, age, gender), and answered some questions about digital devices, and about sleep (number of hours they slept in a normal school day). Then, they filled out different questionnaires in the following order: the PROMIS, and the DASC.

### Data Analysis

For the statistical analyses, SPSS Version 24 was used. First, descriptive statistics of the sample were calculated, taking into account the sociodemographic variables of age and gender.

A confirmatory factor analysis (CFA) was performed following the original validation scale and an existing adaptation in Turkish language (Çimke et al., 2023, Hawi et al., 2019). Given that Çimke et al. (2023) and Hawi et al. (2019) confirmed a two-factor solution comprising an interpersonal and an intrapersonal factor, we sought to find a similar structure in a Spanish children sample.

For reliability, as suggested by the literature (Deng & Chan, 2017), we calculated both alpha and omega coefficients, because the first assumes the potentially unrealistic tau equivalence while the latter does not. Analyses on age invariance and gender invariance were also conducted.

Finally, regarding convergent validity, Pearson correlations were calculated between the Spanish version of the DASC and depressive and anxious symptoms measured by the PROMIS scale.

The data is freely available together with a codebook, the analysis script and the materials of the study in the Open Science Framework repository (see García Morales et al., 2025).

## Results

*Confirmatory factor analysis (CFA).* Under a robust maximum likelihood estimation, our two-factor solution was suboptimal compared to previous efforts and also in terms of traditional benchmarks for model fit (*CFI* = .838, *RMSEA* = .056, *SRMR* = .053). In addition, the standardized correlation between factors was .866, thus deserving a further inspection of the overall factor structure in our sample. Modification indices suggested that correlating four pairs of items would allow to reach an optimal model fit (*CFI* = .903, *RMSEA* = .045 [90%] *CI* [.043, .048], *SRMR* = .046). Specifically, these post-hoc modifications involved correlating items from what was originally planned by the authors as the mood subscale (Items 24 and 5, 266.63), the withdrawal subscale (Items 8 and 21, 136.61, and Items 12 and 21, 80.36), further merged into the intrapersonal factor, and the deception subscale (Items 4 and 16, 74.30), also merged into the interpersonal factor. Given that Item 21 was redundant with two items from the same content, and Item 24 shared a substantial redundancy with Item 8, we chose to drop Items 21 and 24 and ran the analysis again, but model fit did not improve (*CFI* = .901, *RMSEA* = .045 [90%] *CI* [.042, .048], *SRMR* = .045), so we retained our previous solution with 2 factors and 4 pairs of correlated residuals. In Table 1, the loadings of each item on the two factors that composed the DASC can be noted.

**Table 1:** Summary of Statistics for the Spanish-DASC

	Component			
Item	Interpersonal Loading(SE)	Intrapersonal Loading(SE)	*M*	*SD*
4. I lie to my parents about the amount of time I spend using my device (*Miento a mis padres sobre la cantidad de tiempo que paso usando mi dispositivo)*.	.35(.05)		1.22	.67
16. I lie to my parents about what I do on my device *(Miento a mis padres sobre lo que hago en mi dispositivo).*	.37(.04)		1.24	.68
9. My parents try to stop or limit me using my device, but they fail (*Mis padres intentan parar o limitar mi uso del dispositivo, pero no lo consiguen).*	.69(.04)		1.67	1.11
22. I argue with my parents when they ask me to stop using my device (*Discuto con mis padres cuando me piden que deje de usar mi dispositivo).*	.54(.05)		1.38	.81
6. I do not spend time with my family members because I prefer using my device (*No paso tiempo con los miembros de mi familia porque prefiero usar mi dispositivo).*	.44(.05)		1.34	.76
18. I have lost interest in hobbies or other activities because I prefer using my device (*He perdido el interés en mis aficiones u otras actividades porque prefiero usar mi dispositivo).*	.50(.05)		1.35	.81
20. I check my device when I am doing homework or other important things (*Reviso mi dispositivo cuando hago los deberes u otras cosas importantes).*	.57(.04)		2.11	1.30
10. I am sleeping less because I am using my device *(Duermo menos tiempo por estar usando mi dispositivo).*	.54(.05)		1.46	.95
13. I have problems with my parents about the amount of time I spend using my device *(Tengo problemas con mis padres por la cantidad de tiempo que paso usando mi dispositivo).*	.46(.05)		1.35	.78
23. I spend too much money on things for my device *(Gasto demasiado dinero en cosas para mi dispositivo).*	.32(.04)		1.34	.76
25. I continue using my device despite the fact that my grades at school are getting lower and lower (*Sigo usando mi dispositivo a pesar de que mis notas en el colegio son cada vez más bajas*).	.48(.05)		1.44	.90
14. Using my device is the most important thing in my life *(Usar mi dispositivo es lo más importante en mi vida).*	.42(.04)		1.24	.68
17. I am not able to control using my device *(No puedo parar de usar mi dispositivo).*	.75(.04)		1.64	1.01
1. When I am not at school, I spend a lot of time using my device *(Cuando no estoy en el colegio, paso mucho tiempo usando mi dispositivo).*		.69(.04)	2.66	1.22
11. When I do not have my device, I think about what I do on it (video games, social media, and texting, etc.) *[Cuando no tengo mi dispositivo pienso en lo que suelo hacer en el (videojuegos, redes sociales, mensajes de texto, etc.)].*		.79(.04)	2.09	1.28
19. When I stop using my device, it is not long before I start using it again *(Cuando dejo de usar mi dispositivo, no pasa mucho tiempo hasta que vuelvo a usarlo de nuevo).*		.70(.04)	2.11	1.16
5. Using my device helps me to forget my problems *(Usar mi dispositivo me ayuda a olvidar mis problemas).*		.77(.04)	2.54	1.45
15. Using my device is more enjoyable than doing other things *(Usar mi dispositivo es más divertido que hacer otras cosas).*		.64(.04)	1.93	1.06
24. Using my device makes me feel better when I feel bad *(Cuando me siento mal usar mi dispositivo me hace sentir mejor).*		.77(.04)	2.38	1.32
3. I feel upset when I am not able to use my device *(Me siento molesto/a cuando no puedo usar mi dispositivo).*		.82(.04)	1.87	1.16
8. I feel upset when I am asked to stop using my device *(Me siento molesto/a cuando me piden que deje de usar mi dispositivo).*		.82(.04)	1.90	1.12
12. I feel frustrated when I cannot use my device *(Me siento frustrado/a cuando no puedo usar mi dispositivo).*		.80(.04)	1.69	1.06
21. I feel frustrated when I am asked to stop using my device *(Me siento enfadado/a cuando me piden que deje de usar mi dispositivo).*		.70(.04)	1.70	1.03
2. I feel the need to spend more time using my device *(Siento la necesidad de pasar más tiempo usando mi dispositivo).*		.71(.04)	1.88	1.09
7. I have spent more and more time on my device *(Paso cada vez más tiempo con mi dispositivo).*		.77(.04)	1.88	1.06

### Reliability Analyses

Reliabilities for the interpersonal factor are .855 (alpha) and .857 (omega). For the intrapersonal factor the reliabilities are .896 (alpha) and .869 (omega).

### Gender Invariance

In order to show if there is any gender difference in terms of digital addiction, we conducted a measurement invariance analysis, shown in Table 2. A partial scalar invariance was established, with the exception of the noninvariant items DASC7, DASC14, and DASC23 (in other words, these items behave are understood differently by boys and girls, thus are not recommended to gender comparisons). As scalar invariance allows one to compare means across groups, we can observe that the means for intrapersonal and interpersonal factors are significantly lower for girls compared to boys (Interpersonal = -.197, *p* = .003, Intrapersonal = -.261, *p* = .0001).

**Table 2:** Gender and Age Measurement Invariance Models of the Spanish-DASC

Model	χ^2^	*df*	*CFI*	*RMSEA*	*SRMR*	*∆*χ^2^	*∆df*	*p*	*∆CFI*	*∆RMSEA*	*∆SRMR*	Adjustments
Gender												
• Configural	1100.72	536	.91	.046(.043–.049)	0.052							• DASC1 ~~ DASC19 • DASC22 ~~ DASC13
• Metric	1113.26	559	.91	.045(.042–.048)	0.059							
• Scalar	1174.46	582	.9	.046(.043–.049)	0.061							
• Partial scalar	1147.73	579	.9	.046(.043–.049)	0.061							• DASC23 • DASC14 • DASC7
Age												
• Configural	1132.73	540	0.882	.054(.050–.058)	0.055							
• Configural 2	1025.324	532	0.902	.050(.046–.053)	0.053							• DASC9 ~~ DASC10 • DASC15 ~~ DASC24 • DASC1 ~~ DASC19'
• Metric	1080.29	555	0.896	.050(.047–.054)	0.069	54.168	23	0.0002	0.006	0	-0.016	
• Partial Metric	1047.82	552	0.902	.049(.045–.053)	0.062	25.135	20	0.1963	0.000	0.001	-0.009	• DASC11 • DASC8 • DASC23
• Scalar	1169.97	575	0.882	.052(.049–.056)	0.068	157.68	23					
• Partial Scalar	1100.099	572	0.895	.050(.046–.053)	0.064	59.047	20	0.00001	0.007	0.007	0.001	• DASC1 • DASC2 • DASC25
• Partial Scalar 2	1084.81	570	0.898	.049(.045–.053)	0.063	38.19	18	0.003	0.004	0.004	0	• DASC1 • DASC2 • DASC25 • DASC16 • DASC20

### Age Invariance

To evaluate age invariance, we formed two groups of different ages. The first group comprised children between 9 and 11 years, the second group comprised preadolescent between 12 and 14. As can be noted in Table 2, reaching partial invariance came up at the cost of allowing three factor loadings and five item intercepts to be noninvariant across groups. Bearing this in mind, the interpretation of these results has to be done with caution. Although there is no clear rule in the literature for releasing item constraints in nested models, releasing eight items from a 25-item instrument implies that nearly one third of the overall test (i.e., 32%) is noninvariant. With these cautions noted, mean values for the interpersonal component of the DASC, as well as the intrapersonal component were significantly higher for the preadolescent group compared to the children group (.404 and .422 respectively, both *p* < .0001).

### Convergent Validity

Before we can compute correlations between constructs, we assessed model fit for the anxiety and depression constructs, both measured with PROMIS instruments (Cella et al., 2010). Model fit for the anxiety model was deemed excellent (χ^2^ = 1927.056, *df* 28; *p* < .0001; *CFI* = .949; *RMSEA* .069, 90% *CI* [.059–.078]; *SRMR* = .035), while the depression model needed some modifications before it could show good fit (i.e., correlating residuals for Items 2 and 5, and 3 and 4). Therefore, the depression construct model was deemed good (χ^2^ = 285.99, *df* 18; *p* <. 0001; *CFI* = .973; *RMSEA* = .062, 90% *CI* [.062–.053], *SRMR* = .028). With all measurement models calculated, we proceeded to estimate the structural model involving the structural correlation among the Spanish-DASC factors and the anxiety and depression factor. Correlations between intrapersonal-DASC and interpersonal-DASC factors was .872. Correlation among intrapersonal-DASC and anxiety was .505, and with depression was .426. On the other hand, the interpersonal-DASC factor correlated with anxiety and depression in similar way (.493 and .463 respectively).

### Sleep Hours

With respect to sleep, both Interpersonal-DASC and Intrapersonal-DASC showed a negative correlation with hours of sleep (-.100, *p* = .002, and -.150, *p* < .0001 respectively).

## Discussion

The first aim of this study was to examine the psychometric properties of the Spanish version of the Digital Addiction Scale for Children in children aged from 9 to 14 years old. The present study has demonstrated that the DASC-Spanish version is a reliable and valid instrument to assess problematic DD use in children.

Regarding the dimensionality of the DASC, the confirmatory factor analysis conducted in this study proved the bifactorial structure of the DASC-Spanish version. The values explored by the CFI were good, but the two-factor solution was slightly less optimal than that found in previous research (Çimke et al., 2023) as it was found four pairs of correlated items that represent the original subscales of mood symptoms, withdrawal and deception (Hawi et al., 2019). This is in line with most of previous studies about DASC that confirmed the DASC bifactorial structure composed by interpersonal and intrapersonal dimensions (Çimke et al., 2023; Hawi et al., 2019). In this study, factor loadings showed that interpersonal dimension is composed of items that measure the following criteria: deception, conflict, displacement and problems; whereas intrapersonal dimension included those items that analyze mood modification, withdrawal and tolerance; corroborating previous confirmatory analysis (Hawi et al., 2019). It is also noteworthy to specify that the present outcomes also highlighted that the items referred to preoccupation and relapse criteria were included in both dimensions as it was found in the results of the original validation conducted by Hawi et al. (2019).

Furthermore, the high alpha and omega values demonstrated that both interpersonal and intrapersonal dimensions of the DASC-Spanish version exhibited adequate internal consistency. Although the Cronbach’s alpha reported in other validation studies is slightly higher than that found in this study, the present alpha value exceeds the .80 threshold, indicating adequate reliability (Hair et al., 2017). Additionally, this is the first DASC validation study to explore the omega value to assess the instrument’s reliability. Whereas Cronbach’s alpha is based on unrealistic assumptions (individual answers to each item follow a normal distribution, exhibit equal variance and contribute equitably to explaining the underlying factor), McDonald’s omega is independent and exempt from these assumptions demonstrating to be a greater robustness measure of internal consistency than alpha (Stensen & Lydersen, 2022). Thus, the analysis of this value contributes an important advancement to this area of research. The confirmation of the original factorial structure of the DASC, based on two factors, along with adequate reliability supported by a more rigorous statistical measure than alpha, demonstrates that the DASC-Spanish version is a well-founded instrument with strong psychometric properties, making it valuable for use in both clinical practice and research for children aged 9 to14 years.

The findings rejected the third hypothesis of this study, as gender and age differences were indeed found. Girls scored slightly lower than boys on the interpersonal and intrapersonal factors of the DASC. This is consistent with the findings from the validation study of the original DASC scale (Hawi et al., 2019). Different hypotheses support these gender differences in problematic DD. Firstly, previous research has suggested that these gender differences in addictive behaviours may be explained by the traits of impulsivity and self-control may explain differences in addictive behaviours (Mari et al., 2023). Accordingly, females tend to have more self-control and thus, more restraint behaviours, which is negatively linked to addiction. However, males tend to be more impulsive and self-reliant (Charness & Rustichini, 2011), which can lead to higher levels of addiction (Dou et al., 2020). Secondly, online social support, which is a common reason to use DD and social media, is a protective factor for internet addiction in women (Mo et al., 2018). However, non-significant associations were found between social support and internet addiction in males (Mo et al., 2018). In the same line, Tifferet (2020) found in a meta-analysis that females tend to use social media to give and receive social support more frequently compared to males. Receiving online social support may help to regulate emotional difficulties (Mo et al., 2018). Regarding age invariance, significant differences were found between the group of children (ages 9–11) and adolescents. In the validation of the Turkish version of this scale, the same sex and age differences were found (Yılmaz et al., 2023). This can be explained by two reasons. First, the original DASC version has not been validated in adolescents, as it was developed for children (Hawi et al., 2019). Second, as suggested by Tsang et al. (2023), secondary school students use DD more frequently than primary school students (9 to 11 years old). Therefore, future research should explore age invariance only in children, without considering other developmental ranges.

While previous DASC validation studies have explored convergent validity in relation to video game addiction (Yılmaz et al., 2023), the present study analyzed the convergent validity of the DASC using anxiety and depression variables, which are internalizing symptoms that can arise as a consequence of addiction or problematic use. The convergent validity of the DASC-Spanish version was found to be adequate, as positive and significant correlations were observed with symptoms of depression and anxiety measured by the PROMIS scale, thereby supporting the fourth hypothesis. This finding aligns with the study by Hawi et al. (2019), where DASC Global score also correlated with measures of anxiety and depression. The findings suggest that both interpersonal and intrapersonal factors are moderately associated with increased symptoms of anxiety and depression in children. In other words, experiencing greater intrapersonal and interpersonal conflicts related to digital addiction tends to exacerbate internalizing symptoms, namely anxiety and depression. This could be attributed to the tension generated by both intrapersonal and interpersonal conflicts with the DD, combined with the desire to obtain the immediate gratification provided by electronic devices (Wilmer et al., 2017). However, given the cross-sectional nature of this research, causal links cannot be established. In line with compensatory internet use (Kardefelt-Winther, 2014), individuals that are experiencing socioemotional difficulties may use the internet as a maladaptative strategy to cope with symptoms of anxiety and depression. It is important to highlight that whether symptoms of digital addiction may be the cause, or the consequence of other psychopathologies remains unclear (Lozano-Blasco et al., 2022).

The role of digital addiction in anxiety and depression has been well-documented as it is shown by a recent meta-analysis of more than 180 studies, which concluded that lower self-regulation abilities are significantly associated with problematic gaming and smartphone use (Howard et al., 2025). Self-regulation, a critical component of emotional intelligence, is not only influential in substance-related addictions (García-Morales, 2022) but also plays a pivotal role in the onset and progression of mental health disorders (Hairston, 2022), including anxiety and depression. This underscores the importance of further research exploring the role of self-regulation in digital addiction and their influence on mood disorders in children.

The last hypothesis proposed in this study — that higher scores in the DASC would be associated with fewer hours of sleep — was confirmed. Evidence indicates that poorer sleep quality is tied to lower self-regulation, and since lower self-regulation is linked to higher levels of digital addiction (García-Morales, 2022), it follows that digital addiction negatively impacts sleep patterns. This cyclical relationship underscores the need for interventions targeting self-regulation to mitigate the cascading effects of digital addiction on both mental health and sleep quality. The present outcomes specifically show that the increasing of both interpersonal and intrapersonal conflicts arising from digital addiction is associated with reduced sleep duration. Although no research has specifically explored the relationship between digital addiction and sleep patterns in children, previous studies in related areas have demonstrated that increased use of computers and television is associated with fewer sleep hours (Li et al., 2007). These findings align with the current study’s results.

This study is not without limitations, particularly in terms of generalizing the results. First, the participants in the sample are from a single region in Spain, which restricts the broader applicability of the findings. Additionally, the observed gender differences further limit generalizability, as they prevent the assumption that this method of assessing digital device addiction functions equally well across both genders. Furthermore, while comparing participants at two developmental stages — childhood and early adolescence — provides valuable insights, it also complicates a thorough assessment of age invariance. Finally, the cross-sectional nature of this research did not allow to examine causal relationships. Therefore, longitudinal and experimental research focused on the relationships between problematic screen usage, anxiety and depression is needed.

Given these considerations, future research should continue to explore the validity of this instrument in more diverse and contextually varied samples. Problematic use of electronic devices not only differs between children and adolescents but also appears to increase and become more severe with age, as well as in its impact on well-being (Tsang et al., 2023). This highlights the importance of developing reliable preventive programs focused on digital device addiction. While preventive programs have been developed for adolescents (Romero Saletti et al., 2021), those targeting younger children often rely on indirect prevention through psychoeducation and recommendations to parents (Bleckmann et al., 2014).

To provide a foundation for developing preventative strategies that directly target children, it is essential to establish reliable measures for assessing problematic DD use. The Spanish version of the DASC used in this study has proven to be a valid and reliable instrument for this purpose in children, ensuring a solid basis for future prevention programs, as well as interventions for those children with clear addictive symptoms.

## Supplementary Materials

**Table:** 

Type of supplementary materials	Availability/Access
Data	
Study dataset.	García Morales et al. (2025)
Code	
R code.	García Morales et al. (2025)
SPSS Sintaxis.	García Morales et al. (2025)
Material	
Items and translation into English.	García Morales et al. (2025)
Materials.	García Morales et al. (2025)
Study/Analysis preregistration	
The study was not preregistered.	—
Other	
Bookcode codebook.	García Morales et al. (2025)

## Data Availability

The data is openly available together with a codebook in the Open Science Framework repository. These files can be accessed at García Morales et al. (2025).
